# Framing effect, probability distortion, and gambling tendency without feedback are resistant to two nights of experimental sleep restriction

**DOI:** 10.1038/s41598-019-44237-9

**Published:** 2019-06-12

**Authors:** Tina Sundelin, Frida Bayard, Johanna Schwarz, Lukasz Cybulski, Predrag Petrovic, John Axelsson

**Affiliations:** 1 Department of Clinical Neuroscience, Karolinska Institutet, Stockholm, Sweden; 2 Department of Psychology, New York University, New York, NY USA; 3 Stress Research Institute, Stockholm University, Stockholm, Sweden; 4 Osher Center for Integrative Medicine, Karolinska Institutet, Stockholm, Sweden; 5 Division of Psychology and Mental Health, The University of Manchester, Manchester, UK

**Keywords:** Sleep deprivation, Decision, Human behaviour

## Abstract

Several studies suggest that sleep deprivation affects risky decision making. However, most of these are confounded by feedback given after each decision, indicating that decisions may be based on suboptimal feedback-learning rather than risk evaluation. Furthermore, few studies have investigated the effect of sleep loss on aspects of prospect theory, specifically the framing effect and probability distortion. In this within-subjects design, 25 people had (i) two nights of an 8 h sleep opportunity, and (ii) two nights of a 4 h sleep opportunity, in a counter-balanced order. Following the two nights, they performed a gambling task with no immediate feedback; for each round, they could either gamble for a full amount, or take a settlement framed as a gain or a loss for part of the amount. Sleep restriction did not significantly affect the tendency to gamble, the framing effect, or probability distortion, as compared to normal sleep. These results indicate that two nights of sleep restriction affects neither general gambling tendency, nor two of the main predictions of prospect theory. This resilience may be due to a less extreme sleep loss than in previous studies, but also indicates that learning components and risk biases should be separated when assessing the effect of sleep loss on risky behaviour.

## Introduction

Insufficient sleep disrupts complex cognitive processes such as affective regulation, memory consolidation, behavioural inhibition, and executive functions^1–5^–processes which are implicated in risk assessment and behaviour. Most studies suggest that sleep loss increases risk taking and suboptimal decision making in terms of cost-benefit analyses^6–14^, although one study reports a decrease in risky behaviours after sleep deprivation^15^, corroborated only for women^16^, and some report no behavioural differences^12,17,18^, or a slight shift in information use^19^. However, most of these studies have utilized tasks that couple decision making with feedback, making it difficult to dissociate whether insufficient sleep simply induces poor learning from decision-outcome contingencies, or indeed causes shifts in risk preference. If sleep-deprived individuals are worse at registering a negative or positive outcome because they react less to feedback^20^, such feedback might have a smaller effect on future decisions compared to when well rested. In paradigms where feedback is given, well-rested participants may thus be updating their risky choices accordingly, while sleep-deprived participants do not. Indeed, when not relying on feedback, total sleep deprivation did not affect risk seeking^12,18,19^, although prolonged sleep restriction was found to increase risk preference^12^. These discrepant findings are based on many different kinds of tasks and sleep paradigms, often with small sample sizes, making it hard to gauge the conclusiveness of the results.

In cognitive psychology and behavioural economics, risky decision making is often studied through prospect theory, a model that describes humans as more risk-prone when faced with losses than with gains, and having a tendency to under- and overestimate high and low risks respectively^21^. These behavioural tendencies are referred to as the *framing effect* and *probability distortion*^21^. Three studies with gain/loss framing have assessed risk-taking behaviour following sleep deprivation in the absence of feedback. Two support the notion of an “optimism bias”, expressed in terms of elevated gain seeking^9,10^, and one found no effect of sleep deprivation on the framing effect^12,19^. These diverging results are hard to interpret with regard to the aspects of prospect theory, as the first two investigated whether participants maximize gains or minimize losses rather than their propensity for gambling. The third study only used null-hypothesis significance testing, leaving the question open of whether the null finding was due to a lack of effect or a type 2 error.

The neurobiological literature shows that the emotion system of the brain - the amygdala and medial prefrontal cortex (MPFC) - is usually involved in decision biases^22,23^. Increased amygdala activity has been associated with the framing effect, whereas activity in the MPFC predicts reduced susceptibility to framing, as it is associated with an increased rationality index^23^. Furthermore, regulating emotions and amygdala activity through reappraisal reduces loss aversion^22^. These data suggest that prefrontal systems regulate amygdala-dependent decision making as exemplified in the framing effect. Since insufficient sleep has been found to decrease affective regulation, increase mesolimbic activity, and decrease connectivity of limbic systems with the MPFC^1,24^, one would expect sleep-deprived subjects to be more risk seeking under loss frames. This supports the idea that insufficient sleep would lead to a stronger framing effect, paralleling the consequences of poor affective regulation. However, a previous study with one night of total sleep deprivation found no such effects^19^. Recently, it has been argued that the framing effect is more consistent with brain activity during task engagement rather than emotion^25^, with framing-consistent decisions indicating less cognitive effort than framing-inconsistent decisions. Interestingly, this would also suggest a stronger framing effect during sleep loss if the sleep-deprived individual is less attentive and less engaged in the task.

The aim of the present study was to investigate the effect of sleep restriction on gambling tendency and the framing effect in an established decision-making paradigm^23^. In order to not confound risk preference with learning effects, we presented participants with gambles without immediate feedback^23^. We used Bayesian analyses to make informed interpretations regarding potential null effects^26^. An additional aim was to investigate whether insufficient sleep affected probability distortion, illustrated by the tendency to overestimate the value of low-probability gambles^27–29^. We thus restricted participants’ sleep for two subsequent nights, and in a series of gambling choices, participants could either gamble for a full amount or take a settlement. The settlement was framed either in terms of a gain or a loss and was proportional to the probability of winning if gambling.

## Methods

Twenty-five people (13 women) participated in this within-subject study. The subjects were recruited from the greater Stockholm area and had a mean age of 23.5 ± 4.2 years, age range 18–35. They had a self-reported sleep need of 7–9 h/night with no reported health problems or sleep disturbances. As no caffeine was allowed during the study days, heavy coffee drinkers (3 or more cups per day) were excluded. None of the participants were psychology students or had studied psychology; most participants were medical students or not students. They gave written informed consent and received financial compensation for their time. The study was carried out in accordance with the Helsinki Declaration, and approved by the Regional Ethical Review Board in Stockholm, Sweden (Dnr. 2010/1506-31).

### Protocol

Participants came to the lab on two separate occasions in a counter-balanced order: after being instructed to sleep for 8 hours for two consecutive nights, and after being instructed to spend no more than 4 h/night in bed for two consecutive nights. For the 8-hour sleep condition, participants were instructed to go to bed between 22:00 and 00:00 and get up between 06:00 and 08:00, making sure they got 8 hours of sleep. For the 4-hour sleep condition, they were instructed to go to bed between 00:00 and 02:00 and get up no more than 4 hours later, i.e. between 04:00 and 06:00 (for individual bed- and wake-times, see Table S1). Daytime naps and caffeine intake were not allowed during either condition. The lab visits were at least one week apart. Adherence to the protocol was controlled via actigraphs (a watch-like device that measures movement, which is subsequently analysed for sleep/activity. Actiwatch, Cambridge Neuro-Technology Ltd., Cambridge, UK), sleep diaries, and text messages (SMS) sent to the investigator at lights off and waking. Any amount of sleep up to 5 hours on average for the two nights was considered restricted. Participants slept on average 4:00 h less per night during the restricted condition (range 2:53h-5:50 h), see Table 1 for average sleep times (individual sleep times can be found in Table S1). The gambling task was administered between 14:30 and 16:00, keeping the start time constant for each participant between conditions. Following the task, participants rated their sleepiness using the Karolinska Sleepiness Scale (KSS)^30^.

**Table 1:** Average sleep times for the two nights prior to testing.

	Normal sleep		Sleep restriction		t(df)	p
	Sleep time	TIB	Sleep time	TIB		
Actigraphy	8:07 h ± 42 m	8:24 h ± 2:07	4:07 h ± 25 m	4:16 h ± 27 m	29.6 (23)	<0.001
Diary	8:17 h ± 43 m	8:14 h ± 48 m	4:03 h ± 20 m	4:13 h ± 28 m	30.8 (19)	<0.001
SMS	8:03 h ± 19 m		4:08 h ± 16 m		48.1 (16)	<0.001

Mean sleep time and time in bed (TIB) ± standard deviation for the two nights prior testing in the two conditions according to the three different types of reporting. SMS = Short message system sent at lights-off and waking, Diary (Karolinska Sleep Diary) filled out in the morning. T-tests and p-values are for sleep time.

### Gambling procedure

**Figure 1:**
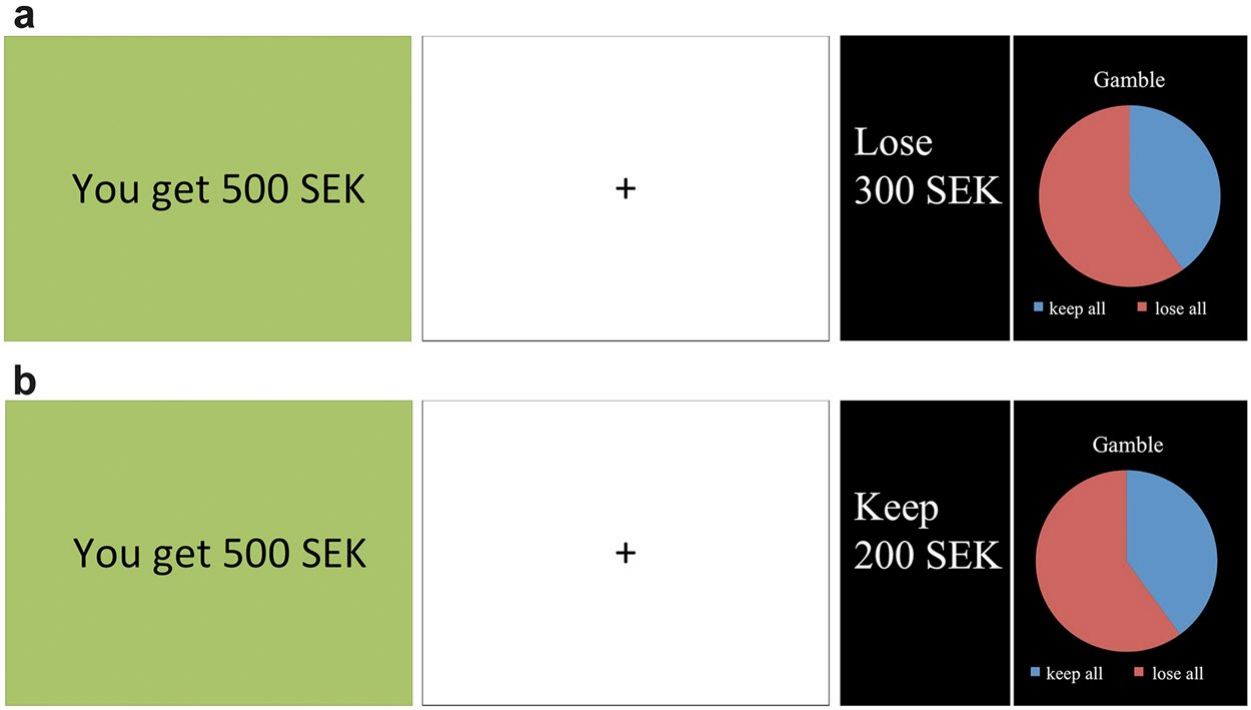
Gambling protocol for (**a**) loss frame and (**b**) gain frame.

Using a well-established paradigm, participants were presented with 96 pictures containing economical information followed by a choice option^23^. The first picture in every round offered the participant a specific sum of money (e.g. “You get 500 SEK”, approximately 55 EUR). In the present study, initial endowments of 250 SEK, 500 SEK, 750 SEK or 1000 SEK were used. The second picture offered the participant a split screen with a safe option, framed as either a loss or a gain (e.g. “Lose 300 SEK” or “Keep 200 SEK” respectively) to the left and a gambling option (e.g. 60% chance of losing the whole pot, 40% risk of winning the whole pot) to the right. If faced with a gamble that had a 40% chance of yielding gain, the participants could either gamble (i.e. 40% chance to win the full sum), or they could take the settlement, which would be 40% of the sum at stake (see Fig. 1 for an example). In other words, the expected value was the same for the safe option and the gamble option. The probability of winning in a given trial was 20%, 40%, 60%, or 80% (apart from the catch trials, see below). In every round, the participant could thus choose between the safe option or gambling, with the same net result in the long run. However, they were not explicitly informed of this net result setup. The choice was made by clicking the left or right mouse button, depending on which side of the screen the chosen option was presented. Each trial option was presented for four seconds, if no choice was made until then the response was registered as a missing value. The percentage of missing values did not significantly differ between the two conditions (normal sleep: 1.4 ± 2.6%, sleep restriction: 1.2 ± 1.4%, z = −0.425, *p* = 0.671). Participants received no feedback on the outcome of their choices^23^.

The paradigm contained 32 positively framed (gain frames) and 32 negatively framed (loss frames) offers as well as 32 control conditions (catch trials). In the latter, the probability in the gamble choice was markedly disproportional to the safe option (e.g. keeping/losing 250 out of 500 but having a 95% chance of keeping the whole sum). The gamble option and the sure option were highly preferable for half of the catch trials respectively. There were two different versions of the paradigm, with different orders of the economical suggestions. The version used was randomized between the two visits, and no participant performed the same version twice. To motivate optimized behaviour, participants were informed that a randomly chosen trial would lead to an actual payout according to their choice (and chance, if they had gambled). This was subsequently paid out along with the study compensation. The main outcome measure was the percent trials gambled. Moreover, reaction time was recorded.

### Analyses

To study the effect of sleep restriction on the framing effect, the percentages of gamble options chosen during gain frames and loss frames were calculated for each individual and condition. A repeated-measures 2 × 2 ANOVA was conducted to determine the effects of sleep condition (normal night sleep/sleep restriction) and frame (loss frame/gain frame). In order to address the effect of sleep restriction on probability distortion, a 2 × 2 × 4 repeated measurement ANOVA was conducted that included the chance to win as an additional factor. Likewise, a 2 × 2 × 4 repeated measurement ANOVA with initial endowment sum as an additional factor was conducted. A 2 × 2 ANOVA was conducted to test the effect of sleep condition and sure versus gamble-weighted gambles in the catch trials. Huynh-Feld correction was applied for *p*-values (uncorrected degrees of freedom reported) and for the ANOVAs, *η*^2^_*p*_ is presented as an effect size.

Mean reaction times were analysed using a 2 × 2 × 2 repeated measures ANOVA with sleep condition (normal sleep/sleep restriction) decision (to gamble/not to gamble), and frame (positive/negative) as factors. The effect of sleep restriction on subjective sleepiness was investigated using a paired t-test. All analyses were conducted using STATA 14 or JASP 2 (Version 0.8.3.1). The statistical significance level was set to *p* < 0.05.

Furthermore, in order to get an estimate of the strength of support for or against the null hypothesis, Bayesian models were estimated for the primary results using JASP 2 (Version 0.8.3.1) with default settings (r scale fixed effects = 0.5). These models were compared to the null model, resulting in Bayes factors (BF_10_ and BF_inclusion_) which can be interpreted as follows: 0–0.3 provides substantial support for the null hypothesis, 0.3–3 means the data are inconclusive, but still provide a direction (below or above 1), and 3-∞ provides substantial support against the null hypothesis^31^.

## Results

### Subjective sleepiness

Subjective sleepiness was significantly higher in the sleep restriction condition compared to the normal sleep condition (mean KSS score ± SD for sleep restriction: 7.6 ± 1.26, and normal sleep: 4.2 ± 1.68, *t*_24_ = −10.1, *p* < 0.001).

### Catch trial analysis

As suggested by De Martino *et al*.^32^, we compared the gambling probability in the catch trials to ensure that subjects were similarly engaged in the gambling task during both conditions. A repeated-measures ANOVA with sleep condition (normal night sleep, sleep restriction), frame (loss, gain) and gamble weighting (sure, gamble weighted) as factors yielded a significant main effect for sure versus gamble-weighted trials (*F*_*1*,2*4*_ = 819.33, *p* < 0.001, *η*^*2*^_*p*_ = 0.972), showing that individuals were very accurate in making optimal choices in the catch trials (Supplementary Fig S1). The main effect of sleep condition and the interactions with sleep condition were not significant (all *p’s* > 0.65), indicating that subjects were responding equally to monetary incentives and were engaged in the task regardless of sleep state.

### Effect of sleep restriction on the tendency to gamble

There was no significant difference in the percentage of gamble options chosen between the normal sleep (M ± SD: 39.8 ± 25.7%) and the sleep restriction condition (M ± SD: 37.1 ± 23.1%) (*t*_24_ = −0.766, *p* = 0.45, Cohen’s d = 0.153, BF_10_ = 0.275).

### Effect of sleep restriction on the framing effect

**Figure 2:**
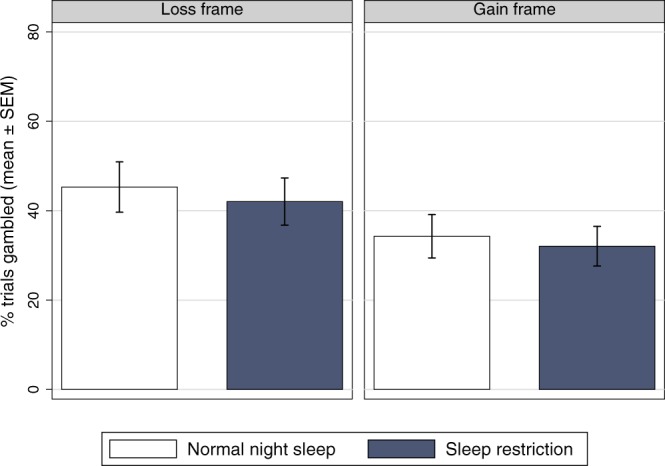
Percentages of trials gambled in the loss frame & gain frame after normal sleep and after sleep restriction to two nights with 4 hours’ time in bed per night. Error bars represent the standard error of the mean (SEM).

As shown in Fig. 2, there was a significant main effect of frame on the percentage of gamble options chosen (*F*_*1*,2*4*_ = 30.47, *p* < 0.001, *η*^2^_*p*_ = 0.559, BF_10_ = 145). Subjects chose the gambling option more frequently in the loss frame (43.6 ± 24.4%) than in the gain frame condition (33.2 ± 21.9%). No difference in gambling was found between the sleep restriction condition and the normal sleep condition (*F*_*1*,2*4*_ = 0.59, *p* = 0.451, *η*^2^_*p*_ = 0.024, BF_10_ = 0.320), and there was no significant interaction between sleep condition and frame (*F*_*1*,2*4*_ = 0.07, *p* = 0.793, *η*^2^_*p*_ = 0.003, BF_inclusion_ = 0.289).

### Effect of sleep restriction on probability distortion

**Figure 3:**
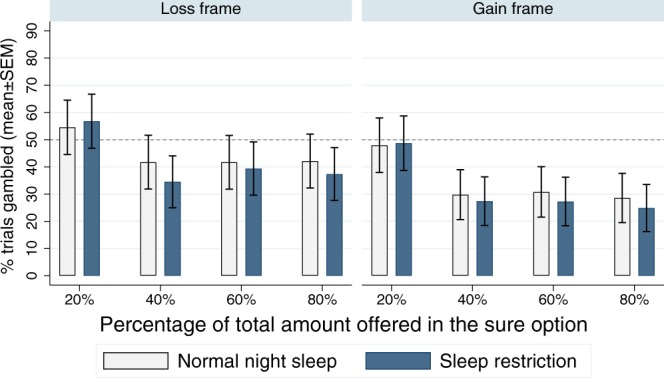
The probability distortion in both sleep conditions for loss and gain frames. Error bars represent the standard error of the mean (SEM). Dashed line represents risk-neutral behaviour.

The repeated-measures ANOVA for percentage of gamble options chosen with sleep condition, frame (loss, gain), and chance to win all, i.e. percent of total amount offered in the sure option (20%, 40%, 60%, 80%) yielded significant main effects for chance to win all (*F*_*3,7*2_ = 4.14, *p* = 0.009, *η*^2^_*p*_ = 0.147, BF_inclusion_ = 73646) and frame (*F*_*1*,2*4*_ = 30.47, *p* < 0.001, *η*^2^_*p*_ = 0.559, BF_inclusion_ = 257) but not for sleep restriction (*F*_*1*,2*4*_ = 0.59, *p* = 0.451, *η*^2^_*p*_ = 0.024, BF_inclusion_ = 0.200). As shown in Fig. 3, subjects were more likely to choose the gambling option with a low percent of total amount offered in the sure option, supporting a probability distortion. None of the interactions were statistically significant (sleep condition by frame: *F*_*1*,2*4*_ = 0.07, *p* = 0.793, *η*^2^_*p*_ = 0.003, BF_inclusion_ = 0.145, sleep condition by chance to win all: *F*_*3,7*2_ = 0.48, *p* = 0.696, *η*^2^_*p*_ = 0.020, BF_inclusion_ = 0.034; frame by chance to win all: *F*_*3,7*2_ = 0.74, *p* = 0.531, *η*^2^_*p*_ = 0.030, BF_inclusion_ = 0.033; sleep condition by frame by chance to win all: *F*_*3,72*_ = 0.37, *p* = 0.773, *η*^*2*^_*p*_ = 0.015, BF_inclusion_ = 0.060).

**Figure 4:**
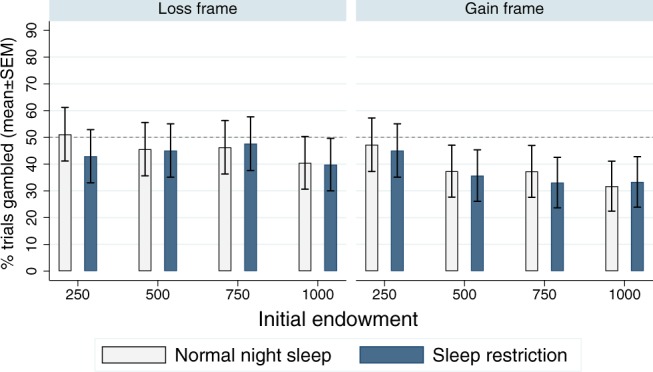
Percentage (%) of trials in which subjects chose the gamble option in the gain and loss frames, for the four different starting amounts for both conditions. Error bars represent the standard error of the mean (SEM). Dashed line represents risk-neutral behaviour.

We also examined the effect of the initial amount offered. Significant main effects were observed for the initial amount offered (*F*_*3,72*_ = 10.11, *p* < 0.001, *η*^*2*^_*p*_ = 0.296, BF_inclusion_ = 1737) and frame (*F*_*1,24*_ = 30.62, *p* < 0.001, *η*^*2*^_*p*_ = 0.561, BF_inclusion_ = 2246000). Moreover, the interaction between frame and initial amount was significant (*F*_*3,72*_ = 6.37, *p* =  < 0.001, *η*^*2*^_*p*_ = 0.210; BF_inclusion_ = 2.80). As shown in Fig. 4, the tendency to gamble decreased with increasing initial amount, although the Bayes factor indicates that this finding is inconclusive. In positive frames, the probability of gambling was particularly high for the initial endowment of 250 SEK. For negative frames, the probability was particularly low for the initial endowment of 1000 SEK. However, there was no significant main or interaction effect for sleep condition (sleep condition: *F*_*1,24*_ = 0.53, *p* = 0.473, *η*^*2*^_*p*_ = 0.022, BF_inclusion_ = 0.360; sleep condition by frame: *F*_*1,24*_ = 0.08, *p* = 0.775, *η*^*2*^_*p*_ = 0.003, BF_inclusion_ = 0.156; sleep condition by initial endowment: *F*_*3,72*_ = 1.40, *p* = 0.251. *η*^*2*^_*p*_ = 0.055, BF_inclusion_ = 0.067; sleep condition by frame by initial endowment: *F*_*3,72*_ = 1.96, *p* = 0.128, *η*^*2*^_*p*_ = 0.076, BF_inclusion_ = 0.182).

### Reaction times

Average reaction times differed between frames (*F*_*1,21*_ = *15.6, p* < *0.001, η*^*2*^_*p*_ = *0.426, BF*_10_ = *0.703)*, indicating that participants took slightly longer to make decisions during negative frames, but that more data is needed for a clear conclusion. There was no difference in reaction time between sleep conditions (*F*_*1*,2*1*_ = 0.016, *p* = 0.902, *η*^2^_*p*_ = 0.001, BF_10_ = 0.166) or between gambling decisions (*F*_*1*,2*1*_ < 0.0.001, *p* = 0.992, *η*^2^_*p*_ < 0.001, BF_10_ = 0.161; see Table S2). There was an interaction between gambling decision and frame (*F*_*1*,2*1*_ = 4.89, *p* = 0.038, *η*^*2*^_*p*_ < 0.189, BF_inclusion_ = 0.459), such that participants were faster in deciding not to gamble during positive frames and slower with this decision during negative frames (Fig. S4). However, based on the inclusion Bayes factor, this is not conclusive. There were no significant interactions regarding reaction time between sleep condition and gambling decision (*F*_*1,21*_ = 2.62, *p* = 0.120, *η*^*2*^_*p*_ = 0.111, BF_inclusion_ = 0.507) or frame (*F*_*1,21*_ < 0.0.001, *p* = 0.983, *η*^*2*^_*p*_ < 0.001, BF_inclusion_ = 0.220).

## Discussion

In this study, monetary risk biases, as defined in prospect theory, were robust against the effects of sleep restriction. Although participants were slightly risk averse in general, neither the framing effect nor the propensity for probability distortion was significantly affected by two nights of sleep restriction - contrary to what might be expected based on the poorer affective regulation found in response to sleep loss^1,24^. Furthermore, contrary to previous research findings^9,10,12^, sleep loss did not affect the tendency to gamble. A main difference between this study and many other sleep-deprivation studies is the focus on risk taking separate from feedback-learning. The increased tendency to gamble in other studies may, at least partly, be due to an impaired ability to learn from previous choices. The findings in this study are in line with three small studies indicating no effect on gambling without feedback following a night of total sleep deprivation^12,18,19^. It would be interesting to discern risk taking from risk learning to see whether risky behaviour with negative outcomes in a real-life setting has less of an impact on future decisions when sleep has been restricted. People who are sleepy may also have a shorter learning span, such that they use more recent information rather than learning from information from longer ago^33^.

None of the previous studies showing increased risk seeking without feedback after a night of total sleep loss included a safe option as an alternative to gambling^9,10^. It is thus possible that when forced to gamble, someone who has not slept chooses a riskier strategy, perhaps due to a reduced ability for cost-benefit analyses, which could be separate from the tendency to gamble per se. We found no effects of sleep restriction on gambling tendency when using a well-established framing paradigm. Strengthening this conclusion, the Bayes factors and observed effect sizes for sleep restriction clearly indicate an absence of pronounced effects of sleep loss. Although a recent review proposed that sleep loss increases risky behaviours^34^, and some have even suggested that sleep loss has “dramatic effects on economic decisions^35^”, our results are more in line with sleep loss having modest or no effects^12,18,19,36^, at least when a safe option is available.

In previous experiments on sleep loss and risky decisions, subjects were deprived of all sleep for 24–75 hours^6–10,12,17–19^, or underwent seven nights of sleep restriction^12^. While extreme conditions of sleep deprivation certainly reflect the reality of some vocations, we emulated circumstances presumably closer to those of the general population - a few nights with restricted sleep. Although one study has indicated this might not be the case^19^, the question remains whether more severe sleep deprivation would have an impact on these facets of prospect theory.

It is widely acknowledged that individuals differ pronouncedly in their vulnerability to sleep loss^37^. In simpler cognitive tasks, this differential susceptibility is usually reflected in terms of different degrees of reaction time variability and accuracy impairment. However, in more complex tasks, such as the decision-making task used in the present study, sleep loss might cause diverging strategy shifts in different individuals; some individuals may display an increased gambling tendency and others a decreased gambling tendency. Different types of motivation may also be a factor. On the one hand, sleepiness could be seen as a motivational state with the purpose of reducing activity and promoting sleep, but on the other hand, if one were trying to stay awake it would be beneficial to engage in alerting activities. Insufficient sleep may thus either reduce the motivation to gamble actively or increase risky decisions to benefit from their alerting effects. Since we found no difference in the tendency to gamble between the two conditions, either neither of these hypotheses hold or the strategy changes cancel each other out. While gambling tendency was relatively stable within individuals across conditions (see Supplementary Fig. S2), there were also individuals showing stronger as well as weaker framing effects following sleep loss (see Supplementary analyses). As task engagement may be an underlying factor in the framing effect^25^, this may further indicate that sleep loss causes divergent strategy shifts in different individuals, or there may be an interaction with amount of sleepiness experienced, such that the most sleepy and the least sleepy take fewer risks^38^. Larger samples are needed to confirm the existence and pattern of such shifts. Although gender differences have been implicated in how sleep loss affects risk biases^13,16^, the current study has insufficient power for such an analysis.

A possible limitation of the present study is that sleep restriction was carried out in the subjects’ homes. However, actigraphy data, sleep diaries, and SMS times all indicate that although some subjects slept slightly longer than the instructed four hours during the sleep restriction nights, the sleep restriction should be seen as successful, and substantial in comparison to what is considered a normal and healthy sleep duration^39^. The increased subjective sleepiness also indicates that the design worked. That being said, although participants were instructed not to, and actigraphy was used, it is possible that subjects took daytime naps without this being registered. It is also possible that participants ingested caffeine despite being instructed not to. The strengths of this study lie in the use of an established paradigm to study gambling probability and the framing effect without introducing learning effects^23^. Both the framing effect and the probability distortion were robust in our data, indicating the validity of the test. The general risk aversion found here has also been shown in previous studies using this paradigm^40,41^. Although the sample size is on the small side, a further strength of the current study is the use of a within-subject design, reducing the risk of confounding factors.

## Conclusions

Two nights of sleep restriction not affect the tendency to gamble, susceptibility to the framing effect, or probability distortion. This resilience to sleep loss contradicts several previous studies, which may be due to them having used rather extreme forms of sleep deprivation, while the present study is based on a more common degree of sleep restriction. In addition, the research field on sleep and risk taking should be seen as young, and hence likely to be affected by a positive publication bias, particularly if true effect sizes and sample sizes are small^42^. Further studies of higher power will have to elucidate which, if any, aspects of risky behaviour are affected by sleep loss, preferably by manipulating sleep loss in a dose-response manner and using tasks that disentangle different components of risk-taking behaviour, such as impulsiveness and learning, as well as looking at non-economic risk taking, and investigating individual and gender differences.

## Supplementary information

Supplementary information

## Data Availability

The datasets generated and analysed during the current study is available from Open Science Framework, https://osf.io/cwv8b.
