# Supplementary material for: Framing effect, probability distortion, and gambling tendency without feedback are resistant to two nights of experimental sleep restriction
Source: Sci Rep. 2019 Jun 12;9:8554. doi: 10.1038/s41598-019-44237-9 (PMC6561965; doi:10.1038/s41598-019-44237-9)
Supplement: Supplementary file 1 — Supplementary information [file 41598_2019_44237_MOESM1_ESM.docx]

**Framing effect, probability distortion, and gambling tendency without feedback are resistant to two nights of experimental sleep restriction**

Tina Sundelin, Frida Bayard, Johanna Schwarz, Lukasz Cybulski, Predrag Petrovic, & John Axelsson

**Supplementary analyses**

In order to display the individual responses to sleep loss, the percentage of gamble options chosen for each individual in the two conditions is displayed in figure S2. To gain a better understanding of whether individuals that would be more likely to gamble in the normal sleep conditions would also be more likely to gamble in the sleep restriction condition, we calculated the Pearson correlation for gambling probability between the rested control and sleep restriction condition. There was a statistically significant correlation between the two conditions (*r* =0.722, *p* < .001), indicating that subjects that had a relatively high tendency to gamble in the normal sleep condition would also have a relatively high tendency in the sleep restriction condition.

In addition, a rationality index was calculated (% gambled in the loss frame trials - % gambled in the gain frame trials) separately for each individual and each condition (normal night sleep/sleep restriction). For the rationality index, a value of 0 represents a complete lack of effect for the framing manipulation indicating that a subject equally much gambles in loss and gain frames. The correlation between the two conditions was not significant for the rationality index (*r* = -.006, *p* = .976, see figure S3) indicating that the effect of sleep restriction on the framing manipulation varied between individuals.

**Supplementary tables**

**Table S1.** Individual sleep times from actigraph data.

| **ID** | **SR** | **Average  bedtime** | **Average  wake time** | **Average  sleep length** | **Average difference  in sleep amount** |
| --- | --- | --- | --- | --- | --- |
|  |  |  |  |  |  |
| 1 | 0 | 23:20 | 07:06 | 07:46 | 03:52 |
|  | 1 | 02:07 | 06:02 | 03:54 |  |
| 2 | 0 | 00:02 | 08:19 | 08:17 | 03:28 |
|  | 1 | 02:16 | 07:04 | 04:48 |  |
| 3 | 0 | 23:19 | 06:46 | 07:27 | 03:20 |
|  | 1 | 06:27 | 10:34 | 04:07 |  |
| 4 | 0 | 23:01 | 06:26 | 07:25 | 03:30 |
|  | 1 | 01:25 | 05:20 | 03:55 |  |
| 5 | 0 | 21:05 | 06:44 | 09:39^1^ | 05:50 |
|  | 1 | 02:07 | 05:56 | 03:49 |  |
| 6 | 0 | 22:51 | 06:40 | 07:49 | 03:20 |
|  | 1 | 01:28 | 05:57 | 04:29 |  |
| 7 | 0 | 23:20 | 07:06 | 07:46 | 03:36 |
|  | 1 | 02:01 | 06:11 | 04:10 |  |
| 8 | 0 | 00:42 | 08:31 | 07:49 | 03:53 |
|  | 1 | 00:57 | 04:53 | 03:56 |  |
| 9 | 0 | 23:26 | 07:49 | 08:23 | 04:33 |
|  | 1 | 02:38 | 06:28 | 03:49 |  |
| 10 | 0 | 23:32 | 07:03 | 07:31 | 03:25 |
|  | 1 | 02:09 | 06:14 | 04:05 |  |
| 11 | 0 | 00:26 | 07:53 | 07:26 | 03:34 |
|  | 1 | 02:12 | 06:04 | 03:52 |  |
| 12 | 0 | 00:05 | 08:01 | 07:56 | 04:02 |
|  | 1 | 01:47 | 05:41 | 03:54 |  |
| 13 | 0 | 23:47 | 07:29 | 07:42 | 03:48 |
|  | 1 | 02:52 | 06:46 | 03:54 |  |
| 14 | 0 | 23:02 | 06:56 | 07:53 | 04:04 |
|  | 1 | 00:59 | 04:49 | 03:49 |  |
| 15 | 0 | 23:20 | 07:11 | 07:50 | 03:52 |
|  | 1 | 01:56 | 05:55 | 03:58 |  |
| 16 | 0 | 00:02 | 08:22 | 08:20 | 04:16 |
|  | 1 | 01:39 | 05:44 | 04:04 |  |
| 17 | 0 | 00:07 | 07:46 | 07:39 | 03:48 |
|  | 1 | 02:11 | 06:01 | 03:50 |  |
| 18 | 0 | 23:03 | 06:48 | 07:45 | 02:53 |
|  | 1 | 00:47 | 05:39 | 04:52 |  |
| 19 | 0 | 23:04 | 08:40 | 09:36 | 05:02 |
|  | 1 | 02:44 | 07:18 | 04:34 |  |
| 20 | 0 | 23:12 | 07:19 | 08:07 |  |
|  | 1^2^ |  |  |  |  |
| 21 | 0 | 23:13 | 07:10 | 08:13^1^ | 04:16 |
|  | 1 | 03:44 | 07:41 | 03:57 |  |
| 22 | 0 | 02:02 | 10:26 | 08:23 | 04:29 |
|  | 1 | 03:24 | 07:18 | 03:54^3^ |  |
| 23 | 0 | 06:17 | 09:20 | 09:03 | 04:46 |
|  | 1 | 03:12 | 07:29 | 04:17 |  |
| 24 | 0 | 23:24 | 07:44 | 08:20 | 04:19 |
|  | 1 | 01:34 | 05:35 | 04:00 |  |
| 25 | 0 | 22:43 | 08:29 | 09:46 | 05:02 |
|  | 1 | 21:20 | 02:05 | 04:43 |  |

^1^Missing data for the second baseline night.

^2^Missing data for both sleep restriction nights.

^3^Missing data for second sleep restriction night.

**Table S2.** Reaction times

|  |  | Mean | Standard deviation |
| --- | --- | --- | --- |
|  |  |  |  |
| All trials | | 1992 | 698 |
|  |  |  |  |
|  | Gamble option | 2007 | 732 |
|  | Safe option | 1982 | 90 |
|  |  |  |  |
|  | Normal sleep | 2025 | 83 |
|  | Sleep restriction | 1958 | 701 |
|  |  |  |  |
| * | Positive frame | 1932 | 699 |
|  | Negative frame | 2051 | 84 |

Reaction times in milliseconds. * Indicates a p-value < 0.05.

**Supplementary figures**

**
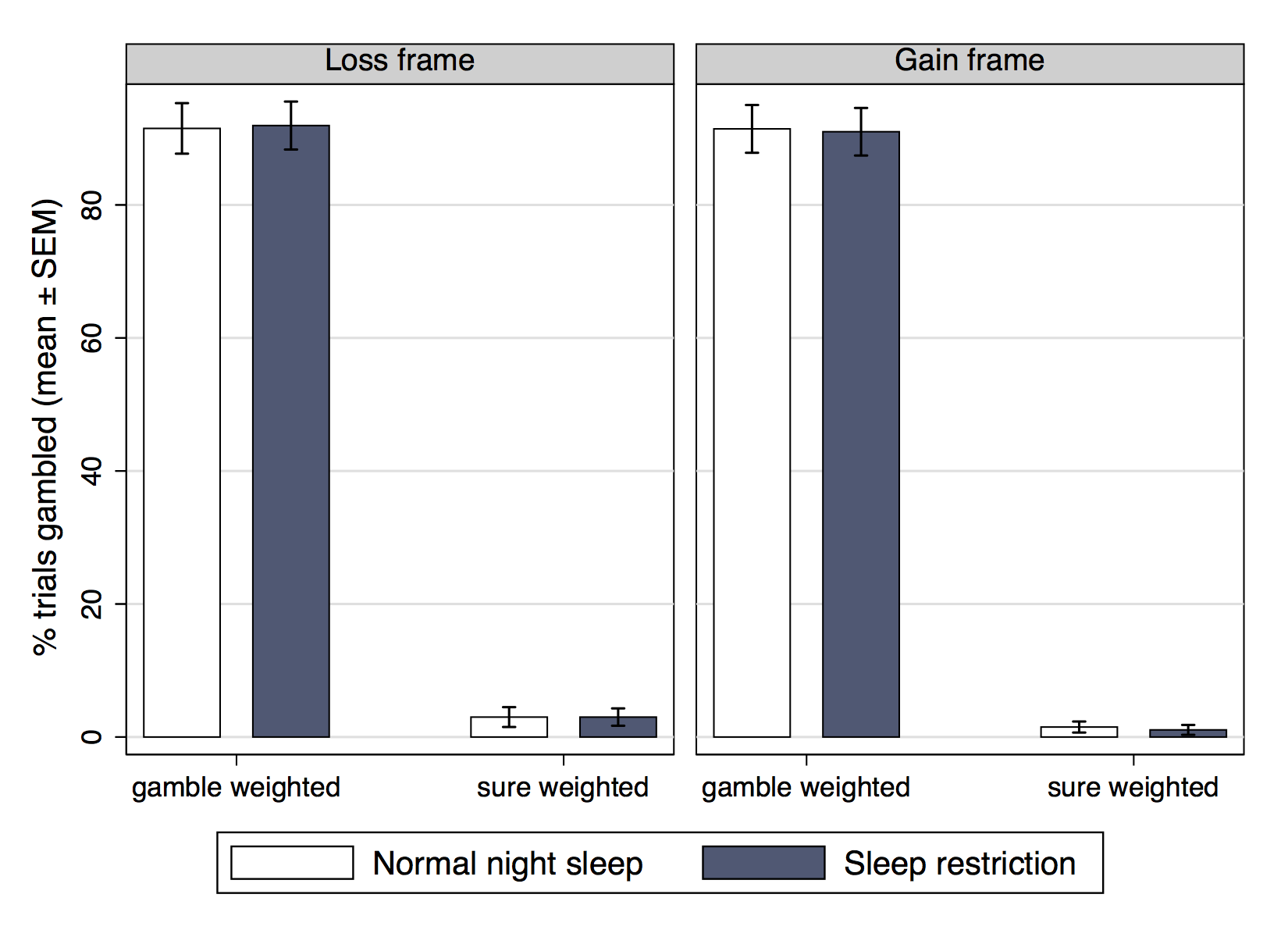
**

**Figure S1.** Results for the catch trials in the normal sleep and the sleep restriction condition for gamble weighted (sure option was 50% of the starting amount and the gamble option was a 95% probability of winning the initial endowment) and the sure weighted (sure option was 50% of the starting amount, the gamble option was a 5% probability of winning the initial endowment) trials.


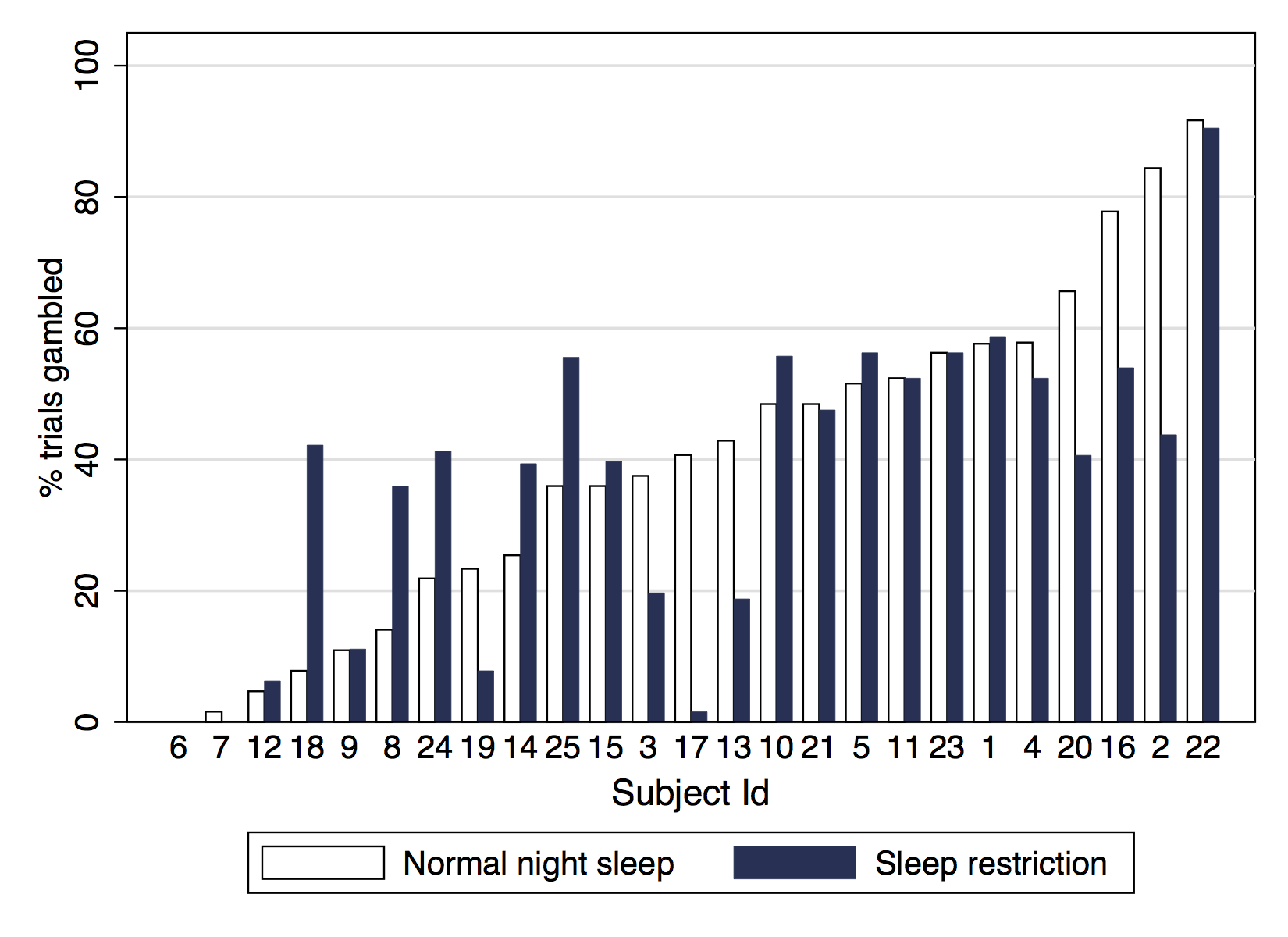


**Figure S2.** Probability to gamble in the two conditions displayed for each subject.


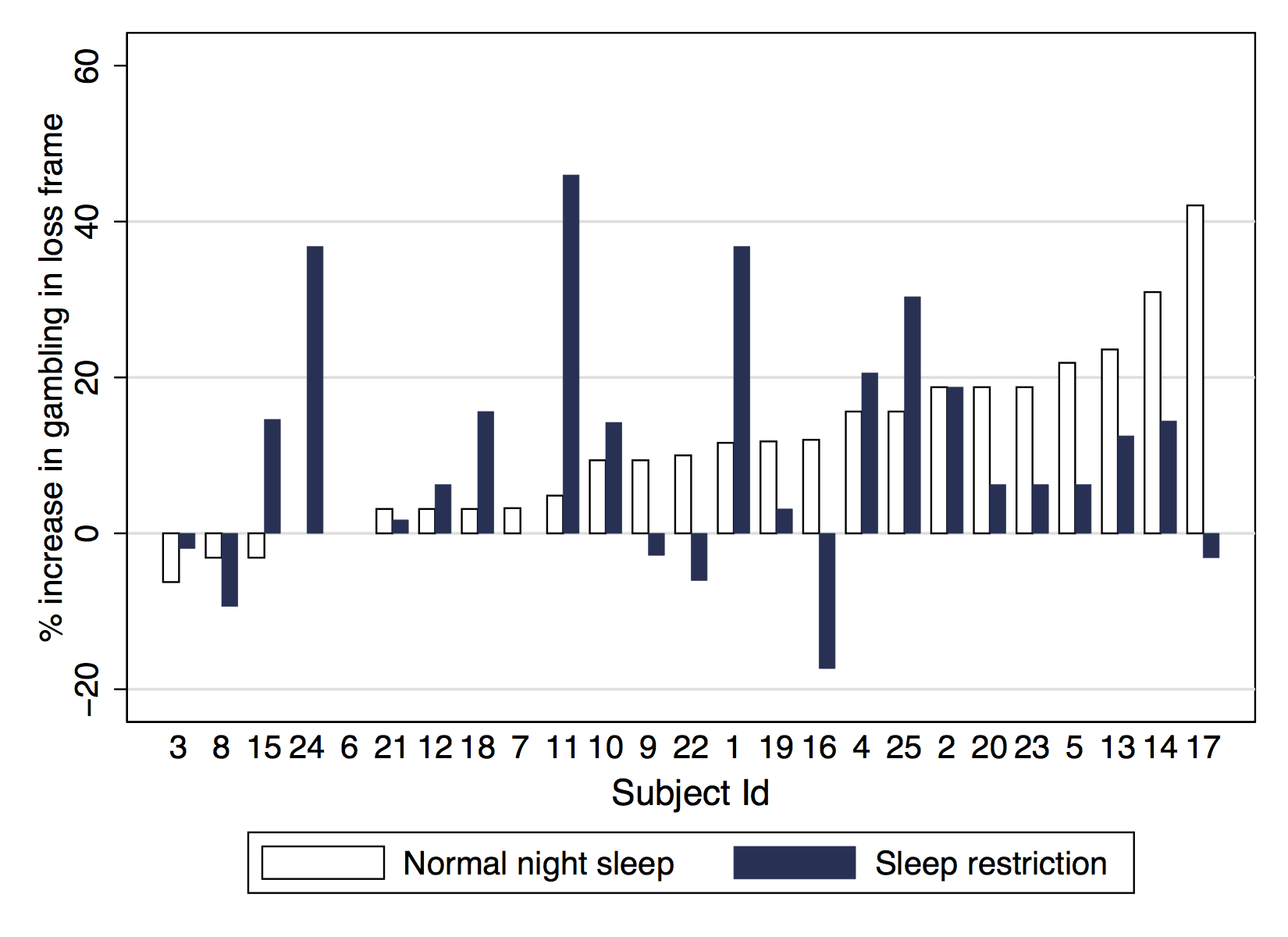


**Figure S3**. Rationality index for each subject in the normal night sleep and the sleep restriction condition.

**Figure S4**. Effects of frame and gambling decision on reaction time.
